# Objective sleep patterns and validity of self-reported sleep monitoring across different playing levels in rugby union

**DOI:** 10.17159/2078-516X/2020/v32i1a8456

**Published:** 2020-01-01

**Authors:** C Leduc, J Tee, P Phibbs, D Read, C Ramirez, T Sawczuk, D Weaving, B Jones

**Affiliations:** 1 Carnegie Applied Rugby Research (CARR) centre, Institute for Sport, Physical Activity and Leisure, Carnegie School of Sport, Leeds Beckett University, Leeds, United Kingdom; 2 Department of Sport Studies, Faculty of Applied Sciences, Durban University of Technology, South Africa; 3 Leinster Rugby, Belfield, Dublin, Republic of Ireland; 4 Yorkshire Carnegie Rugby Union Football Club, Leeds, UK; 5 England Performance Unit, The Rugby Football League, Leeds, UK; 6 School of Science and Technology, University of New England, Armidale, NSW, Australia; 7 Division of Exercise Science and Sports Medicine, Department of Human Biology, Faculty of Health Sciences, the University of Cape Town and the Sports Science Institute of South Africa, Cape Town, South Africa; 8 Leeds Rhinos Rugby League Club, Leeds, UK

**Keywords:** sport, training load, recovery, fatigue

## Abstract

**Background:**

Growing evidence highlights that elite rugby union players experience poor sleep quality and quantity which can be detrimental for performance.

**Objectives:**

This study aimed to i) compare objective sleep measures of rugby union players between age categories over a one week period, and ii) compare self-reported measures of sleep to wristwatch actigraphy as the criterion.

**Methods:**

Two hundred and fifty-three nights of sleep were recorded from 38 players representing four different age groups (i.e. under 16, under 18, senior academy, elite senior) in a professional rugby union club in the United Kingdom (UK). Linear mixed models and magnitude-based decisions were used for analysis.

**Results:**

The analysis of sleep schedules showed that U16 players went to bed and woke up later than their older counterparts (small differences). In general, players obtained seven hours of sleep per night, with trivial or unclear differences between age groups. The validity analysis highlighted a large relationship between objective and subjective sleep measures for bedtime (*r =* 0.56 [0.48 to 0.63]), and get up time (*r =* 0.70 [0.63 to 0.75]). A large standardised typical error (1.50 [1.23 to 1.88]) was observed for total sleep time.

**Conclusion:**

This study highlights that differences exist in sleep schedules between rugby union players in different age categories that should be considered when planning training. Additionally, self-reported measures overestimated sleep parameters. Coaches should consider these results to optimise sleep habits of their players and should be careful with self-reported sleep measures.

Despite the apparent importance of sleep for athletes, growing evidence highlights that elite rugby union players experience poor sleep quality and quantity.^[1]^ The poor sleep experienced by rugby players is of concern given the potential association between low sleep quantity, recovery, injury and performance.

To date, most sleep research in rugby union populations has focused on senior players, and there is a lack of research investigating sleep among adolescent players. Adolescent players are an important population to investigate because they are at a sensitive stage of development. The stress-recovery balance of adolescent athletes is affected by educational (e.g. academic examinations), maturational (e.g. hormonal changes) and social (e.g. pressure to succeed, relationships and peer pressure) demands alongside their sporting requirements.^[2]^ In addition to the unique loads and stresses that adolescent athletes are subjected to, adolescence results in a natural shift to a later chronotype (i.e. diurnal phase preferences) where (given the opportunity) adolescents tend to stay up later at night and sleep later in the morning.^[3]^ Typically, academic and training schedules do not accommodate this later chronotype which may lead to accumulated sleep debt.^[4]^ Therefore, ensuring optimal sleep habits should be a concern for all professionals working with young athletes, however, at present, normative sleep data for adolescent rugby union players is scarce.

Undertaking sleep research in sport is challenging. The majority of sleep research in sport has made use of either objective wristwatch actigraphy monitors or subjective self-reported questionnaires. ^[5,6]^ Actigraphic methods are appealing due to their concurrence with gold standard polysomnography coupled with ease of implementation. However, actigraphy is not an option in many environments due to the cost of the devices or lack of staff with the necessary skills and time for analysis. In addition, actigraphic monitoring protocols are reliant on player compliance and do not always provide an objective measure of sleep quality.

As viable alternative to actigraphy, self-reported sleep questionnaires are low cost and can provide reliable estimates of sleep duration and subjective quality over a longitudinal period. For example, Caia et al.^[7]^ showed that in professional rugby league players, information on sleep duration could be obtained by self-reported measures, but that players overestimated their sleep by 20 min compared to wrist watch actigraphy. The practicality of this method could be extended if the level of agreement of other measures, such as sleep schedule (bedtime and wake-up time) and other derived sleep quality indicators were known. This information would be useful for sport science practitioners who want to monitor sleep on a daily basis with a minimum of constraint for the players, and with a good level of validity.

Therefore, the aims of this study were to investigate and report the habitual sleep patterns of rugby union players, and how they differ between age groups. Secondly, the agreement between a sleep diary and actigraphic measurement was examined. The authors hypothesise that differences in sleep exist between playing levels in rugby union and self-reported sleep data do not reflect objective sleep measures.

## Methods

### Participants

Thirty-eight male rugby union players from a professional club in the United Kingdom were recruited for the study. Nine under U16 (86.1±24.0 kg, 177.9±7.1 cm, 15.7±0.5 years), eleven U18 (88.2±17.1 kg, 182.6±5.9 cm, 17.5±0.7 years), eight Senior Academy (SA) (94.7±13.8 kg, 184.7±9.0 cm, 19.4±0.5 years) and nine elite senior (SE) (106.3±12.0kg, 186.8±10.0cm, 26.2±2.8 years) players participated in this study. Participants were divided in four categories based on their current team. Participants in the U16 age group were 14 to 16 years old, U18 were 16 to 18 years old, and SA players were 18 to 23 years old. All of the players older than 23 years and playing at the full-time professional level were deemed elite senior. Participants provided informed consent prior to the study. Ethics approval was granted by the University’s ethics board and the recommendations of the Declaration of Helsinki were respected.

### Procedure

**Fig. 1:**
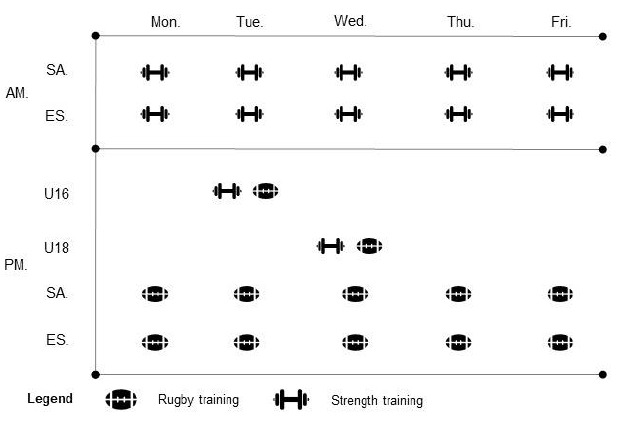
Training schedule for each playing level. For ES and SA gym sessions started at 09:00 and 10:00 for a duration of 60 min while field sessions started at 14:00 and 15:00 respectively. For U18 and U16 gym and field sessions started at 18:00 and 19:00 respectively. SA and ES stand for senior academy and elite senior respectively

Actigraphic sleep assessment was performed on a daily basis to measure sleep quality and quantity across four different age groups (i.e. U16, U18, SA and Elite Senior) for a duration of one week per participant, between July and September 2018. This period corresponded to academic holidays in the UK for U16, U18 and SA players, ensuring that the sleep patterns observed were self-selected and not the result of academic schedules. The training schedule for each playing group is displayed in Fig. 1. Additionally, participants were asked to complete a sleep diary every morning over the same period, as described by Carney et al. ^[8]^

### Sleep assessment

Participants were allocated an Actiwatch MotionWatch 8 (Cambridge Neurotechnology Ltd., Cambridge, UK) which was worn on the non-dominant wrist. Players were instructed to activate the watch by pressing a button before they attempt to sleep, and again after they wake up in the morning. A total of 253 nights were recorded. The sleep variables are presented in Table 1 based on similar methodology used elsewhere (https://pubmed.ncbi.nlm.nih.gov/30789579/).

Sleep patterns were assessed using the Consensus Sleep Diary proposed by Carney and colleagues.^[8]^ Participants were asked to complete the diary each morning on a customised mobile application (Google form, Google, USA). Sleep schedule, total sleep time, sleep efficiency and subjective sleep quality were the variables used. Ratings were recorded in terms of subjective sleep quality using a 5-point Likert scale, where one corresponds to ‘very good’ and five equals ‘very poor’.

### Statistical analyses

Sleep patterns data were investigated using linear mixed models with the playing level as fixed effect and player identity as random effect. Differences between age groups were then assessed with the least squares method test. All the analyses described above were performed with R Studio (Version 1.1.442, R Foundation for Statistical Computing). In an attempt to assess the practical difference of playing level, further analysis was conducted using magnitude-based decisions (MBD). Effect sizes and 90% confidence limits (90% CL) were quantified to indicate the practical meaningfulness of the differences in mean values.^[9]^

Effect size magnitudes were classified as trivial (<0.2), small (>0.2–0.6), moderate (>0.6–1.2), large (>1.2–2.0) and very large (>2.0–4.0). Quantitative changes of greater or smaller changes in sleep parameters were assessed qualitatively as follows: <1%, almost certainly not; 1–5%, very unlikely; 5–25%, probably not; 25–75%, possibly; 75–95%, likely; 95–99.5%, very likely; >99.5%, almost certainly.

Regarding validity analysis, 52 nights were excluded due to the absence of diary responses. In total, 201 nights were analysed. Sleep diary and actigraphy were used as criteria for sleep schedule and sleep quantity respectively. Subjective sleep quality was compared with the fragmentation index and sleep efficiency. The relationship between actigraphy and sleep diary was first examined using a linear mixed model due to the non-independency and repeated measure nature of the data. The sleep schedule, as well as subjective quality validity, were assessed with the self-reported measure as fixed effect, while the sleep duration model was constructed with the wristwatch variables as the fixed effect. To assess the level of agreement, the t statistic obtained from the mixed model was then derived to an r value with 90% confidence intervals (CI). If the 90% CI overlapped positive (0.1) and negative (−0.1) trivial values, the magnitude was deemed unclear. Clear correlations were interpreted as follows: trivial (0.0 to 0.1), small (>0.1 to 0.3), moderate (>0.3 to 0.5), large (>0.5 to 0.7), very large (>0.7 to 0.9) and nearly perfect (>0.9 to 0.1). Further analysis was then conducted in order to obtain mean bias (90% CI), typical error of the estimate (TEE and 90% CI) using a specifically designed spreadsheet.^[10]^ The intercept, as well as the coefficient of the fixed effect, were used to provide a correction equation.

## Results

**Fig. 2:**
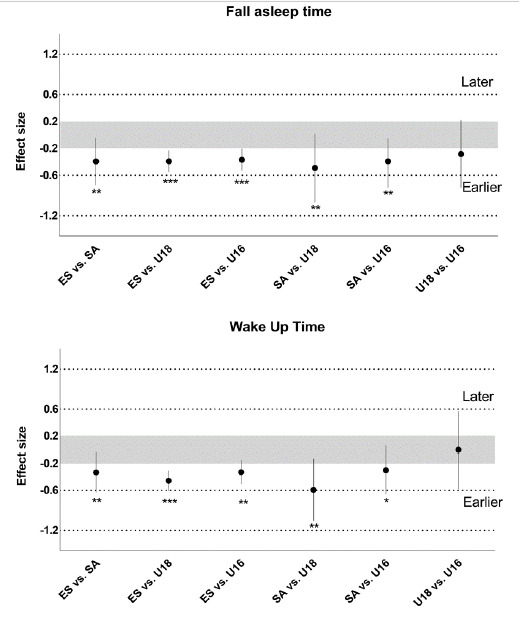
Comparison between categories regarding sleep schedule. *, possibly; **, likely; ***, very likely change/difference between categories. Greys zone stands for trivial. SA and ES stand for senior academy and elite senior respectively. The first playing level mentioned on the x axis designates the order of the comparison (e.g. ES vs. SA: elite senior slept earlier compared to senior academy

The descriptive values of sleep are presented in Table 2. Differences between age groups in terms of sleep schedule are shown in Fig. 2.

For sleep quantity, *possibly* to *likely* trivial differences were observed between elite senior and U16 (0.04 [−0.13 to 0.21]), U18 (−0.05 [−0.24 to 0.14]) and SA (0.11 [−0.22 to 0.45]) players, while other comparisons were deemed unclear. A *possibly* lower sleep quality was observed for SA players compared to U18 (−0.28 [−0.24 to 0.14]). Other results were deemed unclear.

Regarding the fragmentation index, *likely* to *very likely* trivial differences were observed between elite senior, U16 (0.03 [−0.11 to 0.18]) and U18 (−0.05 [−0.29 to 0.19]). A *possibly* trivial difference was found between U16 and SA (0.18 [−0.15 to 0.51]) for the same variable. Other comparisons were deemed unclear. *Likely* better sleep efficiency was found for SA compared with elite senior players (0.49 [0.10 to 0.88]). Other comparisons with elite senior players were deemed unclear. *Possibly* to *likely* worse subjective sleep quality was observed for elite senior players when compared with U16 (0.24 [0.06 to 0.42]) and SA (0.39 [−0.01 to 0.79]) players.

### Validity analysis

Correlations are presented with 90% CI. The results showed a large relationship for bedtime (0.56 [0.48 to 0.63]), and get up time (0.70 [0.63 to 0.75]), with a mean bias of 50.59 min (57.09 to 44.09) and 18.38 min (−9.53 to 27.23) respectively. The correlations for time in bed (0.57 [0.49 to 0.64]) and total sleep time (0.57 [0.49 to 0.64]) also showed a large relationship. Associated mean bias was 87.34 min (79.54 to 95.13) for total sleep time and 77.75 min (69.19 to 86.30) for time in bed. Sleep quality indicators, such as sleep efficiency (0.07 [−0.05 to 0.18]) and the fragmentation index (0.13 [0.01 to 0.24]) showed a small relationship with self-reported sleep quality. Standardised mean bias and typical errors of estimate and calibration equation are presented in Table 3.

## Discussion

The main aims of this study were to i) compare sleep patterns between different age categories of rugby union players and ii) assess the agreement between a sleep diary and actigraphic measures. The current data showed 1) small differences in the sleep schedule between age group categories while total sleep time was consistent across age groups, and 2) the validity analysis highlighted a large typical error and mean bias between self-reported and actigraphy measures. Practitioners should consider these apparent differences in sleep when scheduling training for the different age groups to avoid sleep restrictions. In addition, current study findings suggest precautions should be taken when using subjective questionnaires to report sleep measures.

This study suggests differences in the sleep schedule between playing level with elite senior players, falling asleep and waking up earlier than all other age groups. Caia et al.^[11]^ found similar small to moderate differences between elite senior and junior rugby league players. It is not surprising to observe similar differences in the current study, as later sleep patterns are observed among an adolescent population.^[3]^ Sleep timing is driven by an internal circadian system, a homeostatic drive for sleep and external factors.^[3]^ During adolescence, both internal components (circadian rhythms and homeostatic sleep pressure) change, explaining partially why U16 players slept later than their older counterparts. Additionally, these results can also be explained by psychosocial factors, such as immaturity, social opportunity and independence.^[10]^ The impact of social opportunities on sleep is corroborated by the fact that this study was performed during a holiday period for U16 and U18 players, whilst Senior Academy and elite senior team players were training on a daily basis. This period has been chosen because it allows for the capture of the self-selected patterns of sleep and the avoidance of the effect of the school schedule on sleep patterns. Nevertheless, future studies should investigate sleep patterns when adolescent athletes have educational commitments in order to assess the effect of combined academic and training schedules.

The differences in sleep schedules should be considered by coaches scheduling early training sessions. Early training is common practice for athletes despite a lack of scientific evidence.^[13]^ In support of altering training schedules, early training sessions have been found to restrict sleep and affect performance.^[13]^ Based on this study’s results, when allowed to self-select their sleep schedule, adolescent players woke up at 08:28±01:30 and as such, training should be avoided during this time. Such a scheduling would enable the players to obtain sufficient sleep on a night-to-night basis. Despite constraints related to congested schedules, staff should consider the time between awakening and the first training session in order to optimise sleep and performance.^[14]^

This is the first study to analyse sleep patterns between several age categories in rugby union players. The present results showed only trivial or unclear differences in sleep quantity between age groups. On the whole, the study’s participants achieved the minimum recommended seven hours sleep per night. It should be noted, however, that numerous experts have indicated that athletes may require greater quantities of sleep in order to maintain high levels of performance.^[15]^ Young adults, and young athletes particularly, should aim to achieve nine hours of sleep per night regularly,^[4]^ indicating that further efforts may be required to improve sleep habits in the U16 and U18 age groups.

Athletes can be supported in their need to accumulate additional sleep through sleep extension strategies. It has been demonstrated that when players had the opportunity to extend sleep, it was beneficial for markers of subjective recovery during a training camp.^[16]^ As such, practitioners should consider these results in order to ensure players obtain sufficient sleep quantity.

The findings from the current cohort demonstrate why it is important to monitor sleep in professional and developmental sports settings. Indeed, across the observational period, 14% of the sample experienced at least one night of sleep below the recommended minimum of seven hours. While those recommendations remain debated, monitoring sleep assisted staff to be aware of these poor sleep episodes and to adjust training if necessary, and highlighting the practical usefulness of such approaches. While actigraphic measures are more accessible than polysomnography, it can still be difficult to use such devices on a daily basis, particularly within academy settings, due to human resources and the skills involved by using such devices. Therefore, an agreement analysis was performed in order to assess the validity of self-reported and objective sleep measure.

Despite a strong relationship (0.57 [0.49 to 0.64]) observed between both measures of total sleep time, players tended to overestimate their sleep by approximately 01:30 hours, leading to large standardised typical errors. Such differences might be explained by the fact that total sleep time derived from actigraphy withdraws all the awakenings period that occur during the night, suggesting wrist watch actigraphy is a more sensitive method to capture the real sleep duration among this population. Another factor which could explain these results is the heterogeneity of the population age (range from 16 to 33 years old). This could also explain why trivial and small relationships with sleep efficiency and the fragmentation index were observed. Such a result is not surprising as sleep quality encompasses different sleep dimensions (e.g. issues related to sleep latency, sleepiness, awakenings) which are difficult to summarise within the two objectives.^[7]^ While similar results were found for sleep efficiency by Caia et al.^[7]^ among rugby league players, this is the first study to compare the fragmentation index and sleep quality. When assessed with actigraphy, sleep fragmentation may refer to the amount of movement or restlessness in a sleep period. Such indicators may be helpful in obtaining further insights into sleep quality, which remains difficult using actigraphy. Further comparisons with polysomnography are warranted to confirm the potential use of the fragmentation index as a sleep quality indicator.

While practitioners should consider this inherent error when interpreting the self-reported measure, it is also important to consider the daily constraint that is characteristic to a team sports environment, particularly at the academy level. Indeed, the time, cost and expertise required to collect and analyse actigraphy is an important consideration when working in a fast-paced environment like a team sports club. Based on the present results, the use of actigraphy is encouraged if the actual environment is able to provide human resources fully dedicated to sleep assessment. On the other hand, the use of self-reported measures could be used but practitioners must be aware of the potential bias around using such variables. Correction equations provided in the current study should help practitioners to enhance the accuracy of their sleep data following self-reported measures. Indeed, since short sleep duration has been shown to be related with injury risk,^[4]^ such measures can help practitioners to optimise a player’s safety on a daily basis to align with their fast-paced environment.

### Limitations

Despite the meaningful findings found in this study, a number of limitations exist. The elite senior team performed the study during in season compared to the other teams who were in preseason. Secondly, subgroup analysis, considering a wider range of variables (e.g. travel, match location, match and training timing, social stressors) could be useful in order to perform validity analysis per age group but it is also necessary to understand the differences in sleep patterns. Moreover, the present study was performed in a single club and the conclusions may be specific to this context. Consequently, the generalisation of the present findings remains limited. Finally, a potential limitation of this and other sleep studies is the absence of a specific small worthwhile change for sleep variables. Future studies in this area should consider calculating such values in order to improve decision-making as well as statistical analysis.

## Conclusion

This study showed differences in sleep schedules which have to be considered when early morning sessions are performed within the adolescent categories. Additionally, low sleep quality and quantity were observed without differences between categories. Such results should be considered in order to avoid chronic sleep restrictions which may be of consequence regarding recovery and injury risk. Consequently, it is important to monitor sleep in order to make sure that athletes obtain a sufficient amount of sleep.

## Figures and Tables

**Table 1:** Definitions of each sleep variable from the wristwatch actigraphy

Sleep variables (units)	Definition
**Bed time (hh:mm)**	Estimated clock time at which the player attempts to sleep (press the button marker)
**Fall asleep time (hh:mm)**	Estimated clock time at which the player fell asleep
**Wake time (hh:mm)**	Estimated clock time at which the player woke up
**Get up time (hh:mm)**	Estimated clock time at which the player stop sleeping (press the button marker)
**Time in Bed (hh:mm)**	Time between bed time and get up time
**Sleep onset latency (hh:mm)**	Time between bed time and sleep onset
**Total sleep time (hh:mm)**	Time spent asleep determined from sleep onset to wake up time, minus any wake time
**Wake time after sleep onset (WASO) (hh:mm)**	The total time spent in wake according to the epoch-by-epoch wake/sleep categorization
**Sleep efficiency (%)**	Percentage of total sleep time in relation to time-in-bed
**Fragmentation index (%)**	Sum of the mobile time (%) and the immobile bouts ≤1 min

**Table 2:** Sleep characteristics for the different playing level

	Fall asleep time (hh:mm)	Wake up time (hh:mm)	Time in bed (hh:mm)	Total sleep time (hh:mm)	Wake time after sleep onset (WASO) (hh:mm)	Fragmentation index (%)	Sleep efficiency (%)	Sleep latency (hh:mm)	Subjective sleep quality (AU)
**Under 16**	00:28±01:13	08:28±01:30	08:16±01:46	07:07±01:46	00:53±00:25	29±9	86±5	00:14±00:17	1.91±0.81
**Under 18**	00:11±00:59	08:28±01:07	08:38±01:14	07:22±01:05	00:55±00:28	31±10	86±7	00:18±00:31	2.41±0.84
**Senior Academy**	23:46±00:56	07:51±01:17	08:26±01:07	07:07±01:03	00:58±00:26	32±9	85±7	00:17±00:25	1.97±0.94
**Elite senior**	23:16±00:56	07:10±00:59	08:07±00:59	07:07±00:59	00:47±00:17	31±10	88±5	00:10±00:14	2.53±0.72

Data are presented as mean ± SD.

**Table 3:** Validity analyses between wristwatch actigraphy and the sleep diary

Variables	Means bias (90% CI)	Standardised means bias (90% CI)	TEE (90% CI)	Standardised TEE (90% CI)	Coefficient of correlation (90% CI)	Correction equation
**Bed time (min)**	50.59 (57.09 to 44.09)	0.89 (0.78 to 1.01) Moderate	46.10 (42.62 to 50.26)	1.38 (1.15 to 1.71) Large	0.56 (0.48 to 0.63)	Y=562.90+X^*^0,6245
**Get up time (min)**	18.38 (−9.53 to 27.23)	−0.32 (−0.48 to −0.17) Small	63.52 (58.73 to 69.25)	1.12 (0.95 to 1.35) Large	0.70 (0.63 to 0.75)	Y=80.97+X^*^0.8037
**Time in bed (min)**	77.75 (69.19 to 86.30)	1.19 (1.06 to 1.33) Large	63.91 (59.08 to 69.67)	1.46 (1.20 to 1.83) Large	0.57 (0.49 to 0.64)	Y=275.19+X^*^0.60463
**Total sleep time (min)**	87.34 (79.54 to 95.13)	1.34 (1.22 to 1.46) Large	54.98 (50.83 to 59.94)	1.50 (1.23 to 1.88) Large	0.57 (0.49 to 0.64)	Y=228.19+X^*^0.67238
**Sleep efficiency (%)**	3.52 (2.60 to 4.44)	0.05 (0.04 to 0.07) Trivial	5.83 (5.39 to 6.35)	3.85 (2.62 to 7.10) Very large	0.22 (0.11 to 0.33)	Y=67.47+X^*^0.26034
**Sleep quality vs sleep efficiency**	—	—	—	—	0.07 (−0.05 to 0.18)	—
**Sleep quality vs fragmentation index**	—	—	—	—	0.13 (0.01 to 0.24)	—

Effect size magnitudes were classified as trivial (<0.2), small (>0.2–0.6), moderate (>0.6–1.2), large (>1.2–2.0) and very large (>2.0–4.0). TEE, typical errors of estimate.
